# Estimating Genetic and Maternal Effects Determining Variation in Immune Function of a Mixed-Mating Snail

**DOI:** 10.1371/journal.pone.0161584

**Published:** 2016-08-23

**Authors:** Otto Seppälä, Laura Langeloh

**Affiliations:** 1 Institute of Integrative Biology, ETH Zürich, Zürich, Switzerland; 2 Swiss Federal Institute of Aquatic Science and Technology, Dübendorf, Switzerland; Waseda University, JAPAN

## Abstract

Evolution of host defenses such as immune function requires heritable genetic variation in them. However, also non-genetic maternal effects can contribute to phenotypic variation, thus being an alternative target for natural selection. We investigated the role of individuals’ genetic background and maternal effects in determining immune defense traits (phenoloxidase and antibacterial activity of hemolymph), as well as in survival and growth, in the simultaneously hermaphroditic snail *Lymnaea stagnalis*. We utilized the mixed mating system of this species by producing full-sib families in which each parental snail had produced offspring as both a dam and as a sire, and tested whether genetic background (family) and non-genetic maternal effects (dam nested within family) explain trait variation. Immune defense traits and growth were affected solely by individuals’ genetic background. Survival of snails did not show family-level variation. Additionally, some snails were produced through self-fertilization. They showed reduced growth and survival suggesting recessive load or overdominance. Immune defense traits did not respond to inbreeding. Our results suggest that the variation in snail immune function and growth was due to genetic differences. Since immune traits did not respond to inbreeding, this variation is most likely due to additive or epistatic genetic variance.

## Introduction

Parasites are a ubiquitous selective force [1] against which host individuals defend themselves using mechanisms such as immune system [2]. Understanding the roles of different factors (e.g. physiological, behavioral, genetic and environmental) contributing to variation in host defense traits is highly important for predicting the ecological and evolutionary dynamics of host–parasite interactions. Since adaptive evolution requires phenotypic traits to have a heritable basis [3], genetic variation in host defenses is of particular interest for understanding evolutionary responses to parasite-mediated selection. Several studies have investigated genetic variation in host immune function and parasite resistance by comparing individuals with known relatedness produced through breeding designs [4–11]. These studies have repeatedly reported high family-level variation in host defense traits, thus suggesting their evolutionary potential in natural populations.

Other possibly important factors determining host responses to selection by parasites are non-genetic maternal effects. They occur when the phenotype of the mother affects the phenotype of her offspring in addition to the maternally inherited genes [12, 13]. Since maternal effects alter individual phenotype they can act as alternative targets for natural selection, thereby impacting the direction and rate of evolutionary responses of organisms [14]. In general, maternal effects are common across a wide range of traits and species [12–14], and could also be important for host resistance against infections. This is because many vertebrates [15], as well as invertebrates [16–18] including mollusks [19], can enhance the resistance of their offspring/eggs against parasites by transferring immune factors (e.g. antibodies, alkaloids) to the embryos/eggs.

Estimating the relative importance of direct genetic effects and non-genetic maternal effects in determining phenotypic variation among individuals typically requires complicated breeding designs. A paternal full-sib/half-sib breeding design [20] is most commonly used for this purpose, and also utilized to investigate host defense traits in some species [6, 8–11]. In many ecologically relevant study species, complicated breeding designs are, however, impractical. Therefore, researchers commonly use full-sib families and maternal sibships to approximate genetic variation in defense traits [4, 5, 7, 21, 22]. Those estimates can, however, be confounded by non-genetic maternal effects. Therefore, it would be highly important to understand how much genetic and maternal effects each contribute to the observed family-level trait variation to estimate the suitability of such breeding designs for the conducted research in those systems.

In this study, we examined the relative importance of genetic background and non-genetic maternal effects in determining variation in two immune defense traits [phenoloxidase (PO) and antibacterial activity of hemolymph], as well as in survival and growth, of the simultaneously hermaphroditic freshwater snail *Lymnaea stagnalis*. This species is commonly infected with trematode parasites [23, 24] and increasingly used in ecoimmunological research [25–28]. That work also includes studies in which genetic variation in snail immune function and parasite resistance is investigated using maternal sibships [25, 29] and full-sib families (unpublished data). Since these snails reproduce not only through outcrossing but also through self-fertilization [30] more complicated breeding designs are impractical in this system. Here, we utilized the mixed mating system of *L*. *stagnalis* by producing full-sib families in which each parental snail had produced offspring as both a dam and as a sire. This allowed us to estimate how much genetic background (i.e. family) and non-genetic maternal effects (i.e. dam nested within family) explain of phenotypic variation in the examined traits under standard experimental conditions for this species. Additionally, some snails were produced through self-fertilization. We used those individuals to investigate inbreeding depression which would indicate directional dominance in determining trait variation [31].

## Materials and Methods

### Ethics Statement

This study was carried out in accordance with the laws governing animal experimentation in Switzerland, where work with snails does not require permissions. The study did not involve endangered or protected species. No specific permits were required for the field operations as the used water body is not private property or a nature reserve.

### Study Design

Snails for this study came from a laboratory stock population started from 54 individuals collected from a forest pond in Zürich, Switzerland (47°22’N, 8°34’E), in late June 2012. At the time of collecting, copulations between snails were frequent, and thus we assume that outcrossing rate was high [32]. Furthermore, because *L*. *stagnalis* is able to store allosperm from previous matings and use it for cross-fertilization for over two months after the last copulation [33], the produced offspring of the field collected snails can be considered a good representation of the genetic variation in the wild population. We maintained the lab population in a water tank (temperature: 18 ± 3°C) through mass breeding for eight months (two generations) and fed the snails with fresh lettuce and Spirulina *ad libitum*.

In late February 2013, we haphazardly collected 40 juvenile (shell length < 1 cm) F_2_ generation snails from the lab population, and placed them individually in perforated plastic cups (2 dl) immersed in a water bath (aged tap water, 18 ± 1°C) that was connected to a biological filter. We fed these parental snails with fresh lettuce *ad libitum* and maintained them under these conditions until they started to oviposit self-fertilized eggs. In mid-May 2013, we formed 13 pairs from the parental snails, and placed each pair in a single cup for two weeks to allow snails to mate. We used a longer time period for mating than what has been used in an earlier study by Puurtinen et al. [34] to maximize the probability that each snail mates both as a female and a male. After that, we isolated the snails again and collected the first egg clutch produced during the next seven-day period from each of them. Each clutch marks the individual that laid it as the dam and the snail it was paired with as the sire. These data were recorded for clutches produced by all mating pairs. We placed the clutches individually in 2 dl cups with aged tap water, and changed the water in the cups once a week.

We collected newly-hatched snails from the egg clutches daily, placed them individually in 0.4 dl cups with aged tap water, and fed them with spirulina *ad libitum*. We randomly chose 30 hatchlings from each clutch to be used in the study. We changed the water in the cups twice a week. When the snails were eight weeks old, we measured their shell length to the nearest 0.1 mm using a digital caliper. At that point, we also moved the snails into 2 dl cups, and started to feed them with fresh lettuce *ad libitum*. At the age of 13 weeks (± 1 day), we measured the shell length of each snail that had survived to the end of the study a second time and collected hemolymph samples for immunological measurements (see below). To collect hemolymph we removed the snails from their cups, blot dried them and gently tapped the snail foot until it retreated into the shell, simultaneously releasing hemolymph [35]. We snap froze the hemolymph samples in liquid nitrogen [10 μL of hemolymph mixed with 100 μL of phosphate buffer saline (PBS, pH 7.4) for the PO activity assay, and 70 μL of pure hemolymph for the antibacterial activity assay]. We stored the frozen samples at −80°C. Lastly, we took a tissue sample from the foot of each snail and stored it in ethanol for genetic analyses to test whether the snails were produced through outcrossing or self-fertilization (see below). We could not determine the examined traits from 30 snails, and therefore excluded them from the data.

In a classical full-sib/half-sib breeding design aimed for estimating trait heritability, high replication among families rather than within families is beneficial. In our study, we chose to use a moderate number of families (i.e. mating pairs) but to have a high replication within each family (30 snails per dam per mating pair). We did this because our aim was not to estimate the heritability of the examined traits, but to estimate the potential contribution of non-genetic maternal effects in determining trait variation within families, and thus whether previously described family-level variation in snail defense traits [25, 29] could be due to maternal rather than genetic effects. For this same reason, we chose to conduct the study under standard laboratory conditions rather than to expose the snails to any specific pathogens to activate immune defense. It is important to note, however, that also under these standard laboratory conditions snails are constantly exposed to various opportunistic micro-organisms that invade the snails as a part of the normal exchange between snail hemolymph and the surrounding water [36], which activates their immune function [28].

### Immunological Measurements

We measured PO activity and antibacterial activity of snail hemolymph. PO is a component of the oxidative defenses employed mainly against eukaryotic pathogens [37], whereas humoral antimicrobial proteins are used to resist microbial infections [38]. Both of them are important components of the immune defense in invertebrates including mollusks [39–46]. In *L*. *stagnalis*, these parameters respond to various immune elicitors, and hence are involved in defense against infections [28].

In the PO activity assay, we thawed the stored hemolymph samples on ice and centrifuged them for 15 min at 4000 g at 4°C. After that, we placed 40 μL of the supernatant into a 96-well microtiter plate well containing 140 μL of cold water and 20 μL of cold PBS. We then added 20 μL of cold L-dopa (Sigma-Aldrich, Steinheim, Germany) solution (4 mg/mL in H_2_O) into the well. In the reaction, the enzyme PO oxidizes L-dopa, which leads to an increase in the optical density (OD) of the solution. We measured the OD at 480 nm immediately after adding the L-dopa and again after 6 h incubation period at 30°C using a microtiter plate reader (SpectraMax 190, Molecular Devices, Sunnyvale, CA, USA; instrument photometric range: 0–4 OD with 0 being completely transparent and 4 being non-transparent). The increase in OD is linear during the measurement (O. Seppälä, unpublished data). In addition, we measured six non-hemolymph controls (hemolymph replaced with distilled water) per microtiter plate to control for non-enzymatic oxidation of L-dopa. We calculated the PO activity as a difference in OD between the measurement times (OD after 6 h–OD at time 0; the mean change in non-hemolymph controls was subtracted from the data). We recorded the change in OD in milliunits.

In the antibacterial activity assay, we measured the activity of snail hemolymph against lyophilized *Escherichia coli* cells (Sigma-Aldrich, Steinheim, Germany). We placed 50 μL of a sample into a 96-well microtiter plate well and mixed it with 200 μL of *E*. *coli* solution (0.35 mg/mL bacteria in sodium phosphate buffer, pH 6.0) at 24°C. In the reaction, antimicrobial enzymes destroy bacteria cells, which leads to a decrease in the OD of the solution. We measured OD at 450 nm immediately after mixing the hemolymph with bacteria and again after 30 min using a microtiter plate reader (SpectraMax 190, Molecular Devices, Sunnyvale, CA, USA). The decrease in OD is linear during the measurement (O. Seppälä, unpublished data). In addition, we measured four non-hemolymph controls (hemolymph replaced with distilled water) per microtiter plate to control for changes in OD caused by other factors than antibacterial activity of hemolymph. We calculated the antibacterial activity as a difference in OD between the measurement times (OD at time 0 –OD after 30 min; the mean change in non-hemolymph controls was subtracted from the data). We recorded the change in OD in milliunits.

### Genetic Analyses

We tested whether the individuals in each family were produced through outcrossing or self-fertilization using twelve microsatellite loci (GenBank Accession No. AY225956-AY225959, AY225961-AY225963, EF208747-EF208749, EF208751, and EF208752 [47], Kopp K. C & Wolff K., direct submission to GenBank; see S1 Text for details). We first identified loci at which the parents of each family carried different alleles. We then amplified these loci in ten offspring per family (from one family 8 snails were used as only those survived to the end of the study) and examined if the offspring carried one allele per locus from each parent, which indicated outcrossing. Microsatellite null alleles are known to cause severe challenges for parentage analysis [48]. In our study, null alleles could have led to outcrossed snails appear as homozygous individuals that carry only a maternal allele, thus being interpreted as self-fertilized. This, however, was unlikely because most families interpreted to be self-fertilized could be examined using several (3–6) loci that differed between the parents. In these families, all the used loci consistently supported self-fertilization. One self-fertilized family could be examined using only one locus. However, both parents of this family were heterozygous, and both maternal alleles were observed in the produced offspring indicating that null alleles could not have confounded the result.

### Statistical Analyses

To estimate the role of genetic background and non-genetic maternal effects in determining snail immune function, we analyzed the variation in PO activity and antibacterial activity of their hemolymph using analyses of variance (ANOVAs). We ln transformed PO activity to homogenize error variances, and used models with family (i.e. mating pair) and dam (nested within family) as fixed factors (note that family could not be treated as a random factor as that would not allow nesting of a fixed factor ‘dam’). Since some parental snails reproduced through self-fertilization (see the results section), we excluded all the snails produced by those mating pairs from the analyses as they do not share the same genetic background.

Growth of *L*. *stagnalis* follows a typical power function [49], for which the specific growth rate (ln*S*_2_—ln*S*_1_)/Δ*t* is a linear function of logarithm of size (ln*S*) [where *S*_1_ and *S*_2_ represent size at the beginning and at the end of the time period Δ*t* (5 weeks in our study), respectively, and *S* is their geometric mean] [50]. We did not investigate the growth of snails at the beginning of the study (first 8 weeks) because we could not measure the size of hatchlings. To estimate the role of genetic background and non-genetic maternal effects in determining snail growth, we analyzed the variation in their specific growth rate using a similar model as above except for additionally including ln*S* as a covariate. We analyzed the role of genetic background and non-genetic maternal effects on survival of snails using a generalized linear model with the status of each snail (alive, dead) at the end of the study as a binomial response variable (logit link function) and a similar model as above. For one dam, there was no variation in offspring survival (all individuals survived) so we excluded this mating pair from the analysis to avoid quasi-complete separation that would prevent maximum likelihood estimation.

Since some parental snails reproduced through self-fertilization (based on the genetic analyses, see above), we further tested whether self-fertilized snails differed from outcrossed snails in the examined traits due to inbreeding depression. When self-fertilization occurred it always took place in only one parental snail of a mating pair. Thus, we tested whether self-fertilized snails differed from outcrossed individuals in two separate analyses. First, we analyzed the variation in the examined traits in the snails produced by those parental pairs in which both mating types occurred using models with mating type (outcrossed, self-fertilized) and family (nested within mating type) as factors (ln*S* was included as a covariate for the specific growth rate). Second, we tested whether self-fertilized snails differed from the snails produced by mating pairs in which all the individuals were produced through outcrossing using similar models as above. In the latter analyses, we pooled the individuals from both dams in mating pairs in which all the snails were produced through outcrossing since those snails represented the same genetic background (i.e. family) and did not differ between dams (see the results section). We included ln*S* as a covariate for the specific growth rate.

To investigate if self-fertilization modified the family-level variation in the examined traits, we calculated variances of the family means for each trait in self-fertilized snails, outcrossed snails in the mating pairs in which both mating types occurred, and in snails produced by mating pairs in which both parents reproduced through outcrossing (individuals produced by different dams were pooled as above). We then tested whether these variances differed between self-fertilized and outcrossed snails using *F*-tests for equality of variances. We conducted these tests by comparing self-fertilized snails to outcrossed snails in the same mating pairs, as well as to mating pairs in which all the snails were produced through outcrossing. We performed all statistical analyses using IBM SPSS 23.0 (IBM, Armonk, NY, USA) software.

## Results

**Fig 1:**
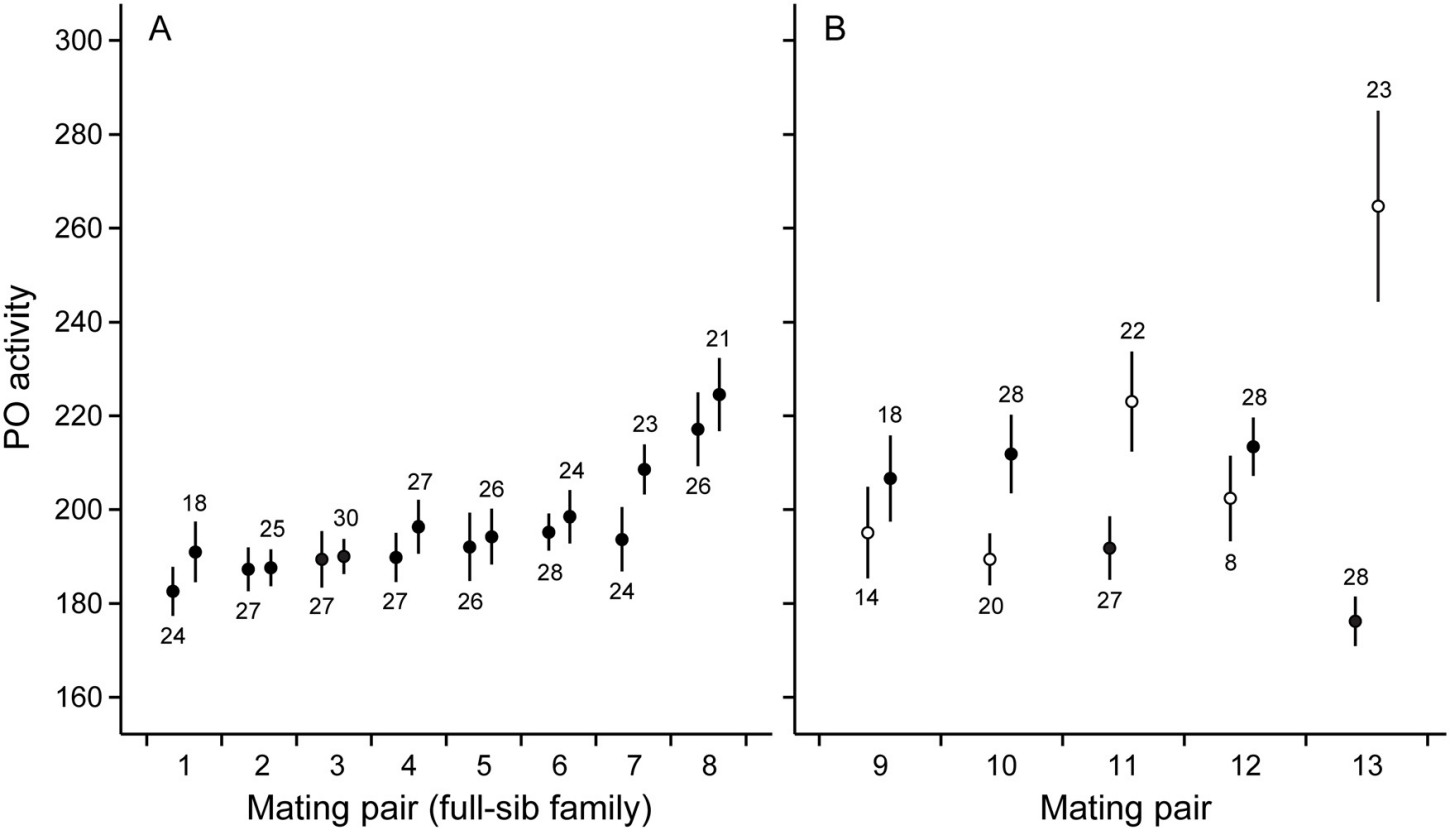
Phenoloxidase (PO) activity (change in optical density over the course of 6 h measured in milliunits) in snail hemolymph. (A) Mean (± SE) of individuals produced by different dams in mating pairs of parental snails in which both dams reproduced through outcrossing (individuals produced by each pair are full-sibs). (B) Mean (± SE) of individuals produced by different dams in mating pairs in which one dam reproduced through self-fertilization (open circles).

**Fig 2:**
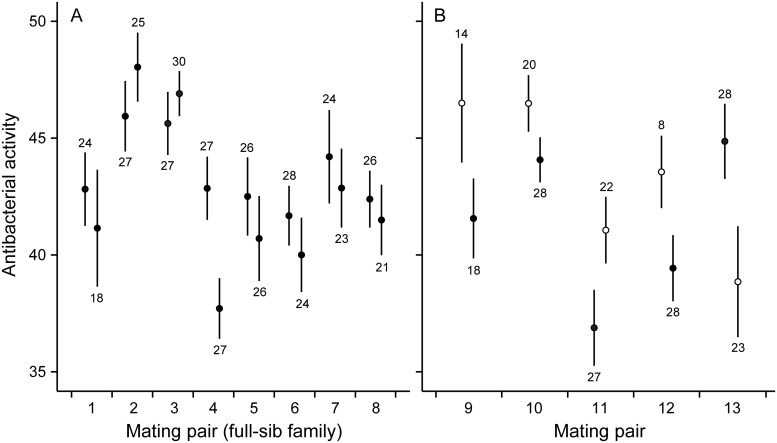
Antibacterial activity (change in optical density over the course of 30 min measured in milliunits) in snail hemolymph. (A) Mean (± SE) of individuals produced by different dams in mating pairs of parental snails in which both dams reproduced through outcrossing (individuals produced by each pair are full-sibs). (B) Mean (± SE) of individuals produced by different dams in mating pairs in which one dam reproduced through self-fertilization (open circles).

**Fig 3:**
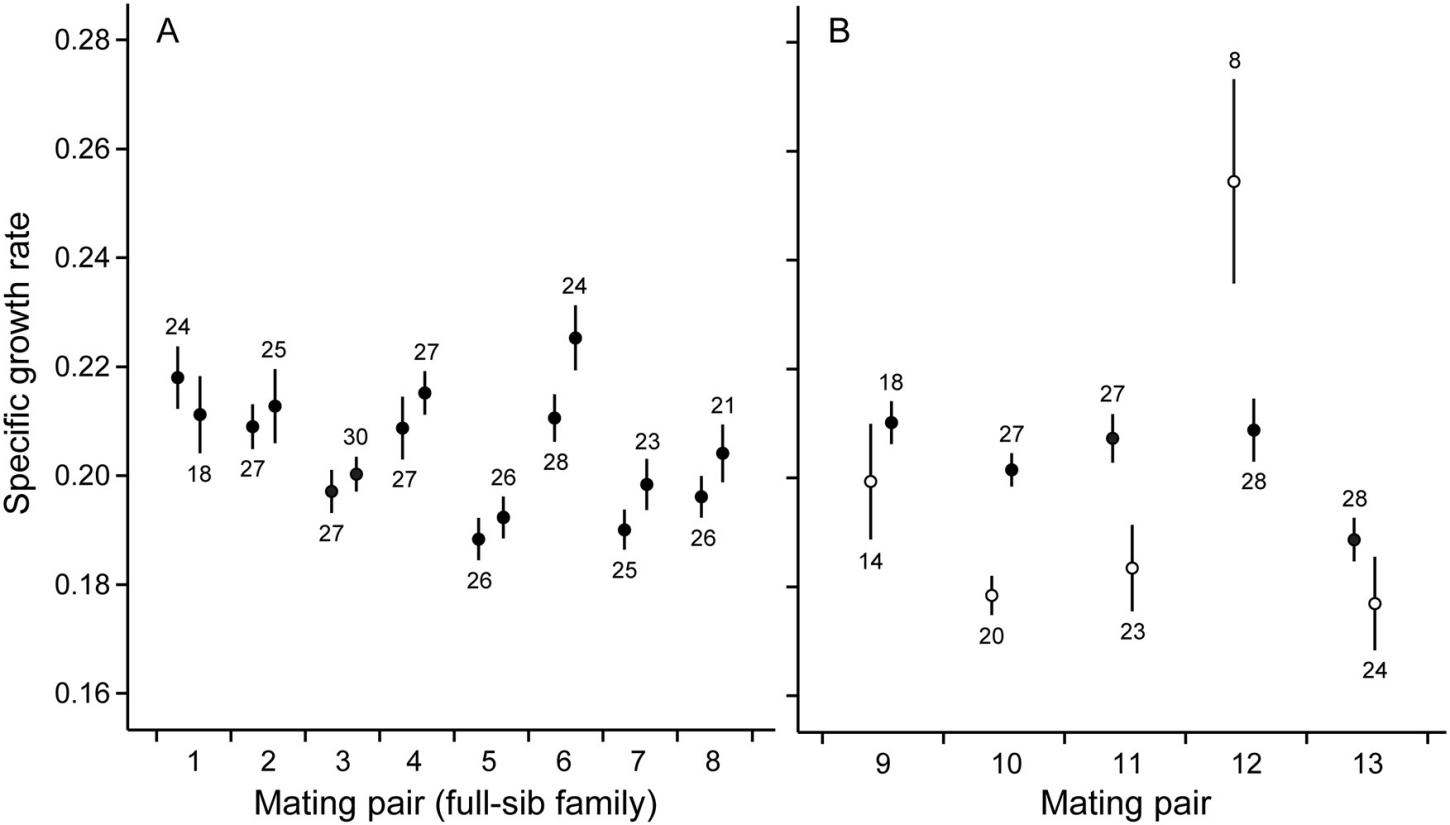
Specific growth rate of snails during the last five weeks of the study. (A) Mean (± SE) of individuals produced by different dams in mating pairs of parental snails in which both dams reproduced through outcrossing (individuals produced by each pair are full-sibs). (B) Mean (± SE) of individuals produced by different dams in mating pairs in which one dam reproduced through self-fertilization (open circles).

**Fig 4:**
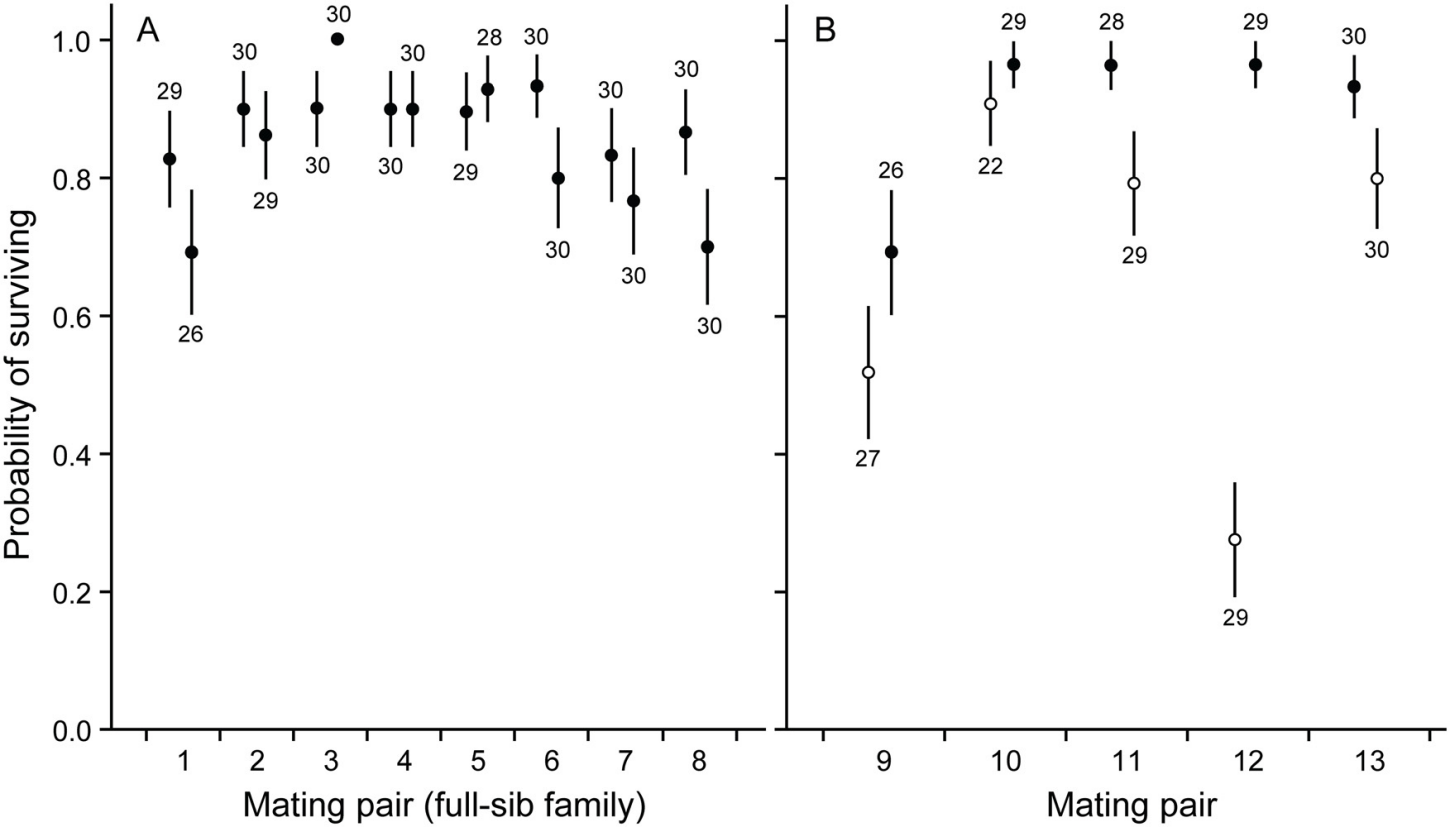
Probability of snails to survive to the end of the study. (A) Probability (± SE) of individuals produced by different dams in mating pairs of parental snails in which both dams reproduced through outcrossing (individuals produced by each pair are full-sibs). (B) Probability (± SE) of individuals produced by different dams in mating pairs in which one dam reproduced through self-fertilization (open circles).

Microsatellite genotyping revealed that in eight mating pairs all genotyped offspring were produced through outcrossing and could be considered as full-sib families. In these snails, family (i.e. genetic background) was a significant predictor for immune defense traits as well as for growth (Table 1, Figs 1a–3a), and explained 8.9–14.1% of total variance in these traits. Which parental snail was the dam of the examined individuals within the families was not a significant predictor of trait variation (Table 1, Figs 1a–3a), and explained only 1.5–2.2% of total variance. Survival of snails was generally high and did not differ among families or dams [generalized linear model: family: Wald *χ*^2^ = 8.692, df = 6, *p* = 0.192; dam(family): Wald *χ*^2^ = 6.612, df = 7, *p* = 0.470; Fig 4a].

**Table 1:** AN(C)OVAs for PO activity and antibacterial activity of snail hemolymph, as well as for specific growth rate of snails during the last five weeks of the study.

Trait	Source	df	MS	*F*	η^2^ (%)	*p*
PO activity	Family	7	0.131	6.345	10.1	< 0.001
	Dam (Family)	8	0.017	0.828	1.5	0.578
	Error	387	0.021			
Antibacterial activity	Family	7	325.973	5.504	8.9	< 0.001
	Dam (Family)	8	71.908	1.214	2.2	0.289
	Error	387	59.228			
Specific growth rate	Family	7	0.005	10.632	14.1	< 0.001
	Dam (Family)	8	0.001	1.189	1.8	0.304
	Size	1	0.029	57.966	11.0	< 0.001
	Error	387	< 0.001			

Factors are family (i.e. mating pair; 8 families) and dam (nested within family). Snail size (ln of geometric mean of initial and final size) was used as a covariate for specific growth rate. η^2^ shows the proportion of total variance explained by each factor. All traits could not be measured from all the individuals.

In five mating pairs, one dam reproduced through self-fertilization. Self-fertilized snails showed slower growth rate and lower survival when compared to outcrossed snails both within the mating pairs in which both mating types occurred as well as when compared to the snails in mating pairs in which all individuals were produced through outcrossing (specific growth rate: ANCOVA: *F*_1,206/479_ ≥ 6.507, *p* ≤ 0.011 for both, Fig 3; survival: generalized linear model: Wald *χ*^2^ ≥ 15.772, df = 1, *p* < 0.001 for both, Fig 4). Self-fertilized snails had higher PO activity when compared to the snails in mating pairs in which all offspring were produced through outcrossing (ANOVA: *F*_1,477_ = 12.397, *p* < 0.001, Fig 1). The same effect was not statistically significant when compared to the outcrossed snails within the same mating pairs (ANOVA: *F*_1,206_ = 3.887, *p* = 0.050). The tendency towards increased PO activity was, however, probably due to one self-fertilized family that showed exceptionally high immune activity. Mating type did not affect antibacterial activity of snail hemolymph (ANOVA: *F*_1,206/477_ ≤ 2.846, *p* ≥ 0.093 for both, Fig 2). The variance of family means in the growth of self-fertilized snails was higher when compared to outcrossed snails (comparison within mating pairs in which both mating types occurred: *F*_4,4_ = 13.626, *p* = 0.013; comparison to mating pairs in which all the snails were produced through outcrossing: *F*_4,7_ = 9.999, *p* = 0.005, Fig 3). Also PO activity and survival showed increased family-level variance in self-fertilized snails when compared to mating pairs in which all the snails were produced through outcrossing (*F*_4,7_ ≥ 7.732, *p* ≤ 0.010 for both, Figs 1 and 4). These effects, however, were not statistically significant when self-fertilized families were compared to outcrossed snails within the same mating pairs (*F*_4,4_ ≤ 4.693, *p* ≥ 0.082 for both).

## Discussion

Our study showed that the family-level variation in the examined immune defense traits, as well as in growth, was likely to arise from genetic differences among families rather than from non-genetic maternal effects. This was demonstrated by the fact that individuals sharing the same parents, but in reversed sex roles, did not differ from each other, whereas individuals with different parents and thus different genetic backgrounds showed high variation. These results indicate that variation in immune function of *L*. *stagnalis* across full-sib families, as well as maternal sibships [25], reflect genetic variation in them well and are unlikely to be confounded by maternal effects. Furthermore, ‘personal’ immune defense expressed by individuals, rather than transgenerational immunity, is likely to be the main target for natural selection in the examined defense traits in our study species. Survival of snails did not show any family-level variation since survival was generally high.

The lack of support for non-genetic maternal effects creating variation in the examined traits is surprising since maternal effects are known to play an important role across a wide range of life-history traits [12–14] as well as in defense against parasites [15–18]. Our study, however, focused largely on performance of adult snails, while maternal effects are most likely to affect offspring performance at early life stages [51, 52]. Thus, our data does not exclude the possibility that the immune function and growth of *L*. *stagnalis* hatchlings is affected by non-genetic maternal effects. Available immunological assays, however, require larger quantities of hemolymph than what we are able to collect from juvenile snails. Similarly, more precise size measurements than currently available would be required for quantifying the growth rate of snail hatchlings. Of the examined traits, survival covers the whole study period. This trait, however, did not show differences that could indicate variation in maternal effects either. Survival of snails was generally high which could have masked the possible role of maternal effects in determining snail performance. Thus, exposure of snails to environmental stress (e.g. resource limitation) could have revealed the possible importance of maternal effects in this trait. It is also important to note that the parental snails were not exposed to any specific pathogens to activate their immune function. Instead, they were maintained under standard laboratory conditions under which they are exposed to opportunistic micro-organisms that invade the snails [36] and activate their immune defense [28]. Furthermore, our analyses could not detect possible variation in maternal investment among eggs within a clutch, which has been described in birds [53].

In the present study, a total of five parental snails reproduced through self-fertilization despite the availability of a mate. Self-fertilized snails showed reduced performance with respect to survival and growth rate when compared to outcrossed snails. This indicates inbreeding depression which is considered to arise from directional dominance where deleterious recessive alleles are expressed in homozygous individuals, or from loss of overdominance [31]. We did not observe inbreeding depression in immune defense traits in *L*. *stagnalis*. It is possible, however, that snails with weak immune function may have died before immune defense traits were measured. Yet our finding is in line with earlier studies in which inbreeding depression has typically not been found in immune function and/or parasite resistance in invertebrates ([34, 54, 55], but see [56]) although such effects are common in vertebrates [57]. When inbreeding depression is not observed, non-directional dominance could still affect trait values. In that case, dominance effects across different loci cancel each other out in determining individual phenotypes [20]. To our knowledge, non-directional dominance variance has been investigated very rarely [58]. However, dominant alleles with deleterious effects are expected to be rapidly eliminated by natural selection, and thus only recessive deleterious alleles can be maintained in natural populations [20]. Therefore, dominance should be directional for fitness related traits. Since immune defense traits are typically under strong selection [59] the genetic variation in snail immune function observed in our study is likely to be additive or epistatic.

Our data also suggest that phenotypic variance among families can increase in self-fertilized snails when compared to outcrossed individuals. The observed increase in family-level variance in self-fertilized snails could take place when the genetic variation is largely additive or there is directional dominance. This is because under random mating, outcrossing individuals having high/low trait values would be most likely to mate with an individual having lower/higher trait values. Thus, outcrossing would lead to offspring phenotypes being closer to the population mean than in the case of self-fertilization. The effects of inbreeding on phenotypic variance due to population bottlenecks have been commonly investigated in earlier evolutionary biological literature [60]. Those studies show mixed results, but also indicate that phenotypic variation can increase after inbreeding [61–64]. Furthermore, matings among siblings have been found to increase phenotypic variance when compared to outcrossed controls [65, 66]. Such effects could significantly contribute to phenotypic trait variation in natural populations of mixed-mating organisms. The change in phenotypic distribution could further alter selection gradients under which traits evolve [66]. In our study system, however, such effects remain to be tested.

To conclude, we found that the family-level variation in immune defense traits and growth of *L*. *stagnalis* snails was largely due to genetic differences among families rather than non-genetic maternal effects. This was demonstrated by the fact that individuals sharing the same two parents (in reversed sex roles) were often similar, but differed from individuals having genetically different parents. Some of the families in this study were produced through self-fertilization, which led to inbreeding depression observed as reduced survival and growth of snails. The examined immune defense traits, however, did not show evidence for inbreeding depression. This indicates that the family-level variation in these immune parameters is likely to be due to additive or epistatic genetic variance rather than due to dominance variance. Thus, full-sib families, as well as maternal sibships that are commonly used in experimental work in this system [25, 29], can be expected to reflect genetic variation and evolutionary potential in parasite resistance well.

## Supporting Information

S1 Dataset Data associated with the analyses performed in this paper. The table has the following columns: mating_pair (pair of parental snails used to produce experimental snails), dam (parental snail that laid the eggs), mating_type [form of reproduction used by the dam (1 = outcrossing, 2 = self-fertilization)], survival [survival of experimental snails to the end of the study (1 = yes, 0 = no)], growth (specific growth rate of experimental snails), ln_size (natural logarithm of geometric mean of initial and final size of experimental snails), PO_activity (phenoloxidase activity of hemolymph of experimental snails), and antibacterial_activity (antibacterial activity of hemolymph of experimental snails).
(TXT)

S1 Text Microsatellite analyses. (DOCX)
