# Supplementary material for: Estimating Genetic and Maternal Effects Determining Variation in Immune Function of a Mixed-Mating Snail
Source: PLoS One. 2016 Aug 23;11(8):e0161584. doi: 10.1371/journal.pone.0161584 (PMC4995018; doi:10.1371/journal.pone.0161584)
Supplement: S1 Text — (DOCX) [file pone.0161584.s002.docx]

**S1 Text. Microsatellite analyses.**

We extracted genomic DNA from the collected tissue samples using Chelex® 100 resin (Sigma-Aldrich, St. Louis, MO, USA). We amplified microsatellites in three multiplex reactions (see Table I), and carried out PCR reactions on TProfessional Thermocyclers (Biometra, Göttingen, Germany). We used the following temperature cycling profile: a 15 min initial denaturation step at 95°C followed by 15 cycles of 30 sec at 94°C, 90 sec at 55°C, and 60 sec at 72°C, followed by 20 cycles with 30 sec at 94°C, 90 sec at 52°C, and 60 sec at 72°C, and a final 30 min extension step at 60°C. We mixed 0.5 μL of each PCR product (1:10 dilution) with 9.3 μL of HiDi^TM^ formamide (Applied Biosystems, Woolston, Warrington, UK) and 0.2 μL of GeneScan^TM^-500 LIZ size standard (Applied Biosystems, Woolston, Warrington, UK) and analyzed them on an ABI Prism 3130xl Genetic Analyzer (Applied Biosystems, Woolston, Warrington, UK). We identified alleles using the GeneMarker software version 2.4 (SoftGenetics, State College, PA, USA).

**Table I Pipetting scheme for microsatellite amplification using three multiplex reactions.**

**References**

1. Walsh PS, Metzger DA, Higuchi R. Chelex 100 as a medium for simple extraction of DNA for PCR-based typing from forensic material. Biotechniques. 1991;10:506-13.
